# Implementation and Effectiveness of Antiretroviral Therapy in Greenland

**DOI:** 10.3201/eid1401.071117

**Published:** 2008-01

**Authors:** Nicolai Lohse, Karin Ladefoged, Niels Obel

**Affiliations:** * Århus University Hospital, Århus, Denmark; † Copenhagen University Hospital Rigshospitalet, Copenhagen, Denmark; ‡ Dronning Ingrids Hospital, Nuuk, Greenland; 1 The Danish HIV Cohort Study: Departments of Infectious Diseases at Copenhagen University Hospitals Rigshospitalet (J. Gerstoft, N. Obel) and Hvidovre (G. Kronborg), Odense University Hospital (C. Pedersen), Aarhus University Hospitals, Skejby (C.S. Larsen) and Aalborg (G. Pedersen), Herning Hospital (A.L. Laursen), Helsingør Hospital (B. Kvinesdal), and Kolding Hospital (A. Møller).

**Keywords:** HIV, Greenland, Polar regions, Arctic regions, Denmark, mortality, highly active antiretroviral therapy, treatment effectiveness, treatment outcome, dispatch

## Abstract

Analyses from the Danish HIV Cohort Study showed that, despite comparable economic means and general education of healthcare personnel, antiretroviral treatment of HIV in Greenland began later and has been implemented at a slower pace with lower therapeutic effectiveness than in Denmark. However, implementation and quality of care improved considerably in recent years.

Like in Western Europe, the first case of HIV was observed in Greenland in the mid-1980s (*1*), but the epidemic in this isolated polar country has evolved differently compared with other industrialized countries (*2*). In a previous study we showed that most patients were infected through heterosexual contact and were middle-aged at the time of diagnosis. Many patients belonged to a socially marginalized group characterized by low income, unemployment, and heavy drinking. Even though highly active antiretroviral therapy (HAART) is tax-supported and free, we found an overall mortality rate of 11% per year for patients given HAART during 1997–2003 (*2*). In a molecular epidemiologic study, we showed that HIV was introduced at least 9 times into Greenland, and that one of these introductions has given rise to a circulating epidemic that has included 76% of all infected persons (*3*). Recently, we found 28% prevalence of transmitted drug resistance, corresponding well with the impression of low drug adherence and high risk behavior (T.V. Madsen et al., unpub. data). Contributing to the disappointing results could be the vast geography with often long distances to healthcare facilities, the short supply of specialized physicians, and the composition of the HIV-infected population. In 2002 the overall responsibility for treating HIV patients was transferred to the Department of Internal Medicine at Dronning Ingrids Hospital, Greenland’s main hospital, located in the capital, Nuuk. The chief physician at that department takes care of HIV patients in Nuuk and supervises treatment of HIV patients in other areas. With access to data from all HIV-infected persons in Greenland and Denmark, we aimed to compare the Implementation and effectiveness of HAART during 1997–2007 in 3 areas: Nuuk; Greenland’s remote districts (all towns and settlements except Nuuk); and the Western European country of Denmark, the former colonial power with which Greenland still has tight economical, social, and constitutional bonds.

## The Study

The population-based Danish HIV Cohort Study (DHCS) collects clinical and paraclinical data on all HIV-infected persons under care in Denmark and Greenland since 1995 (*2**,**4*), including antiretroviral treatment, HIV RNA (viral load), and date of death or emigration. Patients from DHCS were followed from first visit to an HIV clinic until date of death, emigration, or last visit to the clinic. To estimate viral loads and CD4 cell counts between measurements, we carried forward the last observed value. HAART was defined as combination antiretroviral treatment with at least 3 drugs, including at least 1 protease inhibitor (PI), or 1 non-nucleoside reverse transcriptase inhibitor (NNRTI), or abacavir. On January 1 for each year of the study we estimated the proportion of patients receiving HAART, each antiretroviral drug class, and selected antiretroviral drugs. National guidelines in both countries have recommended HAART initiation at a CD4 cell count <300 cells/μL, a threshold that has not changed since 1997. Among patients who had never received HAART, we estimated the proportion with a CD4 cell count <300 cells/μL, and among patients who had begun a HAART regimen at least 90 days previously, we estimated the proportion with a viral load <400 copies/mL. Annual mortality rates (MR) in the HIV population were estimated by person-years analysis; Poisson regression was used to test for trends over time.

## Conclusions

Among 124 HIV patients in Greenland, 98 (79%) were infected through heterosexual contact, 78 (63%) were male, 111 (90%) were Inuit, and 98 (79%) were infected in Greenland. The median age at diagnosis was 50 years (interquartile range [IQR] 40–57 years), and the median CD4 cell count at diagnosis was 350 cells/μL (IQR 220–530 cells/μL). Among 4,702 HIV patients in Denmark, 2,114 (45%) were infected through homosexual contact, 1,745 (37%) through heterosexual contact, and 537 (11%) through intravenous drug use; 3,542 (75%) were male; 3,723 (79%) were Caucasian, and 650 (14%) black African. Half, 2,370, were infected in Denmark, 725 (15%) in Africa, and 1,046 (22%) unknown; the median age at diagnosis was 34 years (IQR 28–42). The median CD4 cell count at diagnosis was 284 cells/μL (IQR 108–490 cells/μL).

**Figure 1:**
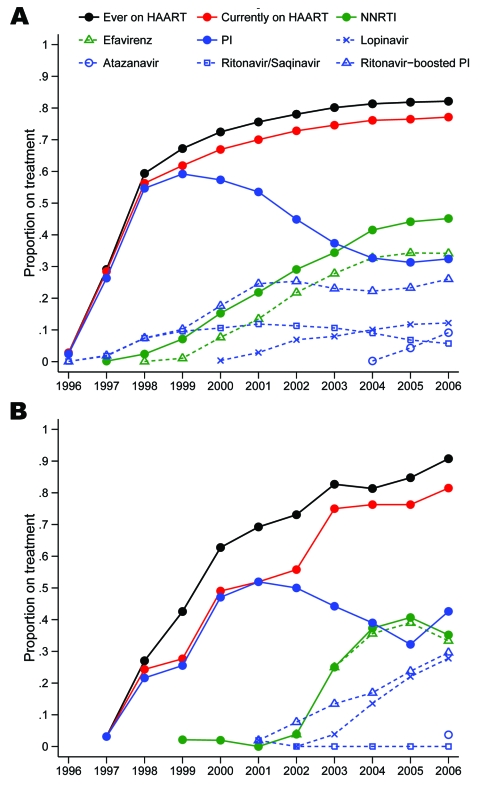
Proportion of patients receiving treatment on January 1, 1996–2006 in A) Denmark and B) Greenland. Numerator, patients who were receiving antiretroviral therapy as part of a HAART (highly active antiretroviral therapy) regimen. Denominator, all patients under observation. NNRTI, non-nucleoside reverse transcriptase inhibitor; PI, protease inhibitor.

In Greenland only 3% had begun HAART on January 1, 1997, as opposed to 28% in Denmark (Figure 1). The proportion on HAART increased gradually up to 81% in 2006, but not until 2003 did the proportion in Greenland reach the level in Denmark. Further, as late as 2001, 96% of all treatment regimens in Greenland included an unboosted PI (26% in Denmark), and in 2002 only 7% were NNRTI based (40% in Denmark). At that time the International AIDS Society USA guidelines carefully encouraged the use of boosted PIs, and NNRTI-based regimens were considered an equally effective alternative to PI-based regimens (*5*). Only after 2002 did the pattern shift, and in 2006 it was similar to that in Denmark, with approximately half of the combinations being NNRTI based, the other half PI based, with ritonavir-boosted lopinavir used in 65% of all PI regimens on January 1. The newer PI atazanavir was used in only 9% of PI regimens in 2006 (28% in Denmark). NNRTI used in Greenland has almost exclusively been efavirenz, whereas in Denmark 24% of NNRTI use on January 1, 2006, was nevirapine. The difference between the curves “ever on HAART” and “currently on HAART” in Figure 1 reflects the number of persons currently interrupting their treatment; the proportion with interruption is higher in Greenland than in Denmark. Structured treatment interruptions have not been recommended in Greenland or Denmark, so these persons supposedly have interrupted their therapy because of compliance problems. There was no difference in the uptake of HAART between Nuuk and the remote districts in Greenland (data not shown).

**Figure 2:**
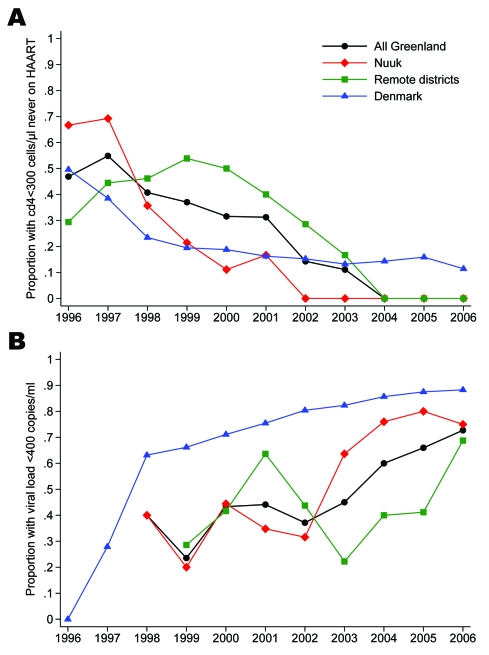
A). Proportion of patients with low CD4 cell count on January 1, 1996–2006. Numerator, patients with a CD4 cell count <300 cells/μL. Denominator, all patients who had not yet been prescribed a highly active antiretroviral therapy (HAART) regimen. B) Proportion of patients with suppressed viral load on January 1, 1996–2006. Numerator, patients with an HIV RNA <400 copies per mL. Denominator, all patients who had ever started a HAART regimen.

Until 2002, >30% of patients not yet receiving HAART in Greenland had a CD4 cell count <300 cells/μL (Figure 2). In comparison, the proportion in Denmark has been <30% since 1998, with <5% having a CD4 cell count <200 cells/μL since 2001. Among patients ever starting a HAART regimen, the proportion with suppressed viral load in Greenland was <45% until 2003 but has increased to 73% in 2006 (Figure 2). Nuuk reached the 75% mark in 2004, whereas the increase in the remote districts started later and reached 69% in 2006. The proportions in Denmark were 62% in 1998, 81% in 2003, and 88% in 2006.

The overall mortality rate among HIV patients in Greenland decreased from 139 (95% confidence interval [CI] 81–239) per 1,000 person-years in 1995–1997 to 59 (95% CI 35–99) in 2004–2006, corresponding to a 9% decrease per year (mortality rate ratio [MRR] = 0.91, 95% CI 0.84–0.98, p = 0.014) (Table). The decrease was most marked in patients in Nuuk (MRR = 0.86, 95% CI 0.77–0.96, p = 0.006) and less in the districts (MRR = 0.96, 95% CI 0.86–1.08, p = 0.533).

**Table:** Mortality rate per 1,000 person-years among HIV patients in Greenland and Denmark, 1995–2007*

Variable	Greenland			Denmark
	All	Nuuk	Remote districts	
Years				
1995–1997	139 (81–239)	178 (89–356)	103 (43–247)	96 (88–105)
1998–2000	82 (47–144)	62 (26–148)	107 (51–225)	29 (25–34)
2001–2003	84 (50–141)	107 (58–199)	54 (20–144)	25 (22–28)
2004–2006	59 (35–99)	43 (19–96)	81 (41–162)	24 (21–28)
Mortality rate ratio change per year, 1995–2006	0.91 (0.84–0.98)	0.86 (0.77–0.96)	0.96 (0.86–1.08)	0.82 (0.80–0.84)
p value†	0.014	0.006	0.533	<0.001

*Ranges in parentheses are 95% confidence intervals. †Test for trend.

Treatment of HIV patients in Greenland began at a later stage of disease and has been implemented at a slower pace with lower therapeutic effectiveness than in Denmark, despite comparable economic means, general education of healthcare personnel, and common therapeutic guidelines. From other studies we know that patient support and education improve adherence (*6*) and that guideline-recommended therapy is more likely to be chosen if the physician is specialized in HIV and has >20 HIV patients in care (*7*), regardless of whether this physician is a generalist or infectious disease specialist. We observed marked improvements in the choice of antiretroviral drug combinations and effectiveness of HAART from 2003 onwards. These advances coincided with the establishment of a dedicated team in Nuuk and were most pronounced in that city when compared with the remote districts. Even though this temporal association does not prove causation, the improvements are likely to be partly attributable to the increased focus on HIV in the capital. The MR among HIV patients in Nuuk in recent years was higher than that in Denmark, but part of this difference may be attributable to a high background mortality rate among HIV-uninfected persons in Greenland (*2*,*8*) and an older HIV-infected population. As previously reported, sexually active persons in Greenland undergo frequent HIV testing (*2*), and CD4 cell counts were high at diagnosis, ruling out late testing as a contributor to the high MR. In conclusion, healthcare systems in the sparsely populated and isolated polar areas may be less fit to take on state-of-the-art care and treatment for HIV or other diseases previously unknown in the area, and an extra effort from the such providers may be needed to maximize control of the disease.
